# Post-traumatic stress disorder amongst children aged 8–18 affected by the 2011 northern-Namibia floods

**DOI:** 10.4102/jamba.v8i2.169

**Published:** 2016-01-13

**Authors:** Simon Taukeni, George Chitiyo, Morgan Chitiyo, Ina Asino, Genesia Shipena

**Affiliations:** 1 School of Health Sciences, University of Fort Hare, South Africa; 2 College of Education, Tennessee Technological University, United States; 3 Department of Counseling, Psychology and Special Education, Duquesne University, United States; 4 Office of Career Development, University of Namibia, Namibia; 5 Office of Student Affairs, University of Namibia, Namibia

## Abstract

Extreme flooding in the northern parts of Namibia occurred in 2011, impacting many school-going children in the region. The rationale for the current research is to assess post-traumatic stress disorder (PTSD) on school children as a result of the floods. A self-administered Child Trauma Screening Questionnaire (CTSQ) with closed-ended questions was administered to 480 children between the ages of eight and 18 years at their respective schools. The CTSQ consists of five items assessing re-experiencing and five items assessing hyper-arousal symptoms. The results show that 55.2% of learners aged 12 and below and 72.8% of learners aged 13 and above reported experiencing symptoms of trauma from the floods 2 years after the event. These percentages were quite high and are therefore a cause for concern. Given the magnitude of this problem, it is important for the government and other stakeholders to provide the necessary psychological and/or emotional support in the event of future floods or similar disasters.

## Introduction

Disasters, whether natural or man-made, seem to occur indiscriminately around the world. Veenema and Schroeder-Bruce (2002) define disasters as events which result in the disruption of essential services such as communication, education, health delivery and sanitation. Such events include floods, earthquakes, droughts, wars and diseases. Wherever they occur, disasters tend to leave devastation and anguish amongst the affected populations (Chitiyo, Changara & Chitiyo 2008; Chitiyo & Chitiyo 2009; Gangi & Barowsky 2009; Shibley 2010).

In Africa, both natural and man-made disasters continue to wreak havoc across countries. Whilst the HIV and AIDS epidemic continues to devastate communities, other natural disasters such as floods, wildfires and droughts also affect countries across Africa (Lukamba 2010). Whereas much scholarly attention has been given to HIV and AIDS and its impact on African communities, it seems that little research has been done on the impact of – and response to – other disasters such as floods. Southern Africa is one of the regions that have been devastated by floods in recent years. A chronological analysis of disasters in southern Africa over the past 150 years indicates that floods and droughts are amongst the most common disasters in the region (Southern Africa Environment Outlook 2008). In March 2000, for example, cyclone El Nino caused severe flooding that left at least 900 people dead in Mozambique, which was the hardest hit country (Lukamba 2010). Based on the chronological analysis of the Southern Africa Environment Outlook (2008), it is predicted that southern Africa will continue to experience such disasters (Lukamba 2010).

Namibia has experienced flooding in some areas of the country in recent years with the worst floods reported in 2011. According to the International Federation of Red Cross and Red Crescent Societies (2011), the north-western and north-eastern parts of Namibia have been experiencing the worst flooding in decades since the beginning of January 2011. The six regions affected are Kunene, Oshana, Omusati, Ohangwena, Oshikoto, Kavango and Caprivi. Nearly 500 000 people were affected with over 60 000 displaced, approximately 19 000 in relocation camps and 65 related deaths reported as a result of the disaster. The Oshana region has a population of 161 916 of which 7.4% (*n* = 11 922) were severely affected by the floods and needed assistance in the form of tents, canoes, food and medication. Twenty-five people died from drowning in Oshana (Shivute 2011). Many of the people affected by the 2011 floods had not fully recovered from the 2008 and 2009 floods. Families and their school-going children were severely affected. In 2009, about 69% (83 out of 131) schools were affected whilst 63% (*n* = 15 301) of learners affected.

When excessive flooding occurred in 2011, the local communities were not well prepared for a disaster of this magnitude, resulting in severe impact on the locals. In addition to the physical impact that children in the Oshana region faced due to the flooding, it stands to reason that they were also affected emotionally and that some may actually have post-traumatic stress disorder (PTSD) due to the lingering impact of the disaster. Children are more susceptible to the impact of disasters as noticeable in many complex psychological and behavioural symptoms.

The impact on Namibian children have not been studied before. There is a void in the literature about the impact of flooding on school children and the adequacy of the response efforts in the affected areas such as the Oshana region. This current study intends to fill this gap.

## The impact of disasters

The impact of disasters is not only physical but include psychological scarring and persistent emotional trauma (Chitiyo & Chitiyo 2009). According to Shibley (2010), the impact of disasters on school-age children includes somatic complaints, re-experiencing, arousal as well as disruptive behaviour. It is also not unusual for older children to experience depression and disillusionment (Shibley 2010). The National Child Traumatic Stress Network has documented evidence of the effects of different kinds of disasters on families and children. With specific reference to floods, the most frequent reactions of people that have been exposed to floods include increased feelings of insecurity, anxiety, disruptive behaviour, irrational fears (phobias), disturbances in sleep or appetite, somatic symptoms such as stomach aches or headaches and decreased school performance (National Child Traumatic Stress Network n.d.).

One of the most important aftermaths of disaster on children is PTSD. Studies conducted in several places around the world where disasters had struck documented cases of PTSD (Veenema & Schroeder-Bruce 2002). Children who suffer from PTSD will have a limited ability to function in social groups, including family. They also exhibit signs of school phobia and a decline in academic performance. It was, therefore, one of the goals of this study to ascertain the extent to which the children were affected so that appropriate intervention services may be recommended.

There is dearth of literature addressing the psychological impact of flooding on children, particularly in Namibia and in southern Africa in general. For this reason, this study was an attempt to address this void. Although this study was conducted 2 years after the disaster occurred, we feel that it will be an important contribution towards understanding the difficulties that children go through when disasters occur, particularly when their psychosocial needs are not attended to sufficiently and in a timely manner. For effective learning to take place, children have to be emotionally stable and their physical needs met.

## Problem statement

Floods appear to be a recurrent natural disaster in southern Africa with research predicting continued future occurrences (Lukamba 2010). Unfortunately, the impact of floods on communities can be quite devastating. Apart from the sudden death and displacement of people and the loss of property, floods can also have long-term consequences such as diseases, food shortages and economic instability for the affected communities (Buzdar & Ali 2011; Defra 2004). It has also been established that when such disasters strike, children are amongst the most vulnerable and usually experience the physical, emotional and psychological impact of the disasters (Chitiyo *et al.* 2008). According to Save the Children (2011), most parents are unable to deal with their children’s changed behaviour after the floods, and as a result, children suffer from behavioural and psychological problems, including anxiety, depression and phobias. Oshana region experienced severe flooding in 2011 which left families and communities displaced, resulting in the loss of livelihood, lives and property. As a result, basic services such as schools and clinics were disrupted. Many villages were inaccessible and cut off from the road networks. Some schools had to close down, and community members were relocated to higher ground.

Following the 2011 floods, the government of Namibia conducted a post-disaster needs assessment. Using the damage and loss assessment methodology, the main focus of which is on the overall economy of the affected country and the household level, the assessment reports on the devastating impact of floods in the northern parts of the country in different sectors, including the environment, agriculture, industry, tourism, housing, health and education. These factors had an impact, either directly or indirectly, on children. For example, many families were left without access to basic needs such as food, shelter and education. The report states that some of the school children’s needs were not met and that their traumatic experiences were not addressed. If left unaddressed, these traumatic experiences can have devastating long-term consequences on the children’s education as discussed by Chitiyo *et al.* (2008). There is no guarantee that the floods will not re-occur in the future. It is, therefore, necessary to explore the psychosocial impact that the floods had on school children. In light of this background, the specific purpose of this study was to investigate the psychosocial impact (specifically PTSD) of the floods on school-going children between the ages of 8 and 18 years. This age range was chosen to include children who were at least 6 years old during the time of the flooding, the assumption being that those less than six at the time of the floods would not be able to accurately remember details of the disaster. The hypothesis to be tested was that a majority of the children, particularly the younger ones, would report signs of PTSD as assessed by the Child Trauma Screening Questionnaire (CTSQ).

## Methodology

Data for the study were obtained through a survey of learners using the self-administered CTSQ. A total of 484 learners were requested to take part in the survey. Of this number, 89.5% (*n* = 433) completed the CTSQ. The remaining 55 either did not give their assent to participate or their parents did not sign the consent form. The sample came from 14 schools that at the time of the flooding were either camping (meaning that they had a different, temporary location), closed, suspended or intended to camp. To ensure the representativeness of the sample, schools were selected so that all parts of the five educational circuits of the region that were variously affected were included in the sample.

The purpose of the CTSQ is to screen children, who have been exposed to disasters, for trauma. Adapted by Kenardy, Spence and Macleod (2006) from the popular Trauma Screening Questionnaire (TSQ) to make it suitable for children, the CTSQ assesses re-experiencing (5 items) and hyper-arousal symptoms (5 items). The instrument has item-total correlations ranging from 0.14 to 0.50 and reasonable internal consistency reliability (α = 0.69) (Kenardy *et al.* 2006). Permission to use the CTSQ was obtained from the developers of the instrument. The original instrument was adapted to suit the specific Namibian situation. Specifically, the instrument’s wording was revised to refer precisely to the flooding in Namibia, and it was also translated into the vernacular Oshiwambo for students who might not have a good command of English, especially the younger ones.

Authorisation to conduct research was granted in writing by the Ministry of Education through the Office of the Permanent Secretary. School inspectors, principals and parents of school children who participated were informed about the study in writing in the form of a letter which contained information about the purpose of the study as well as the researchers’ contact information. The letter also guaranteed the respondents’ anonymity and confidentiality and stated that participation was voluntary. Parents were requested to sign the consent letter as an indication that they allow their children to take part in the study. Learners without signed consent letters were not allowed to participate in the study. Learners whose parents had consented to them participating were also asked to give their own verbal assent.

The researchers ensured that participation in the study did not involve risk of physical or psychological harm to the participants. The data in this article do not contain personally identifying information. Special care was provided to young learners as they relived their experiences of the floods. The researchers collecting data were professional social workers and educational psychologists with skills to observe and detect any signs of behavioural concerns relating to children’s conduct during the data-collection process. No incidents of visible psychological trauma were recorded.

The CTSQ is usually used to screen children for trauma, usually in a short period following the occurrence of a disaster, so that the children can receive specialist services. This was not the case with this study. Extreme flooding in the northern parts of Namibia occurred in 2011, and the screening tool was administered in 2013, 2 years later. The primary motivation for doing this assessment was to determine whether the services that were provided to school children following the floods were adequate to help them cope with the effects of the disaster. The researchers’ intent was to use the CTSQ to simply reveal the possible magnitude of the problem of trauma experienced by children due to floods and to determine the extent to which the response to the disaster was sufficient in addressing the psychosocial needs of the children. Depending on the extent of the problem, appropriate action may need to be taken to address the psychosocial needs of children in the affected communities.

## Results

**FIGURE 1:**
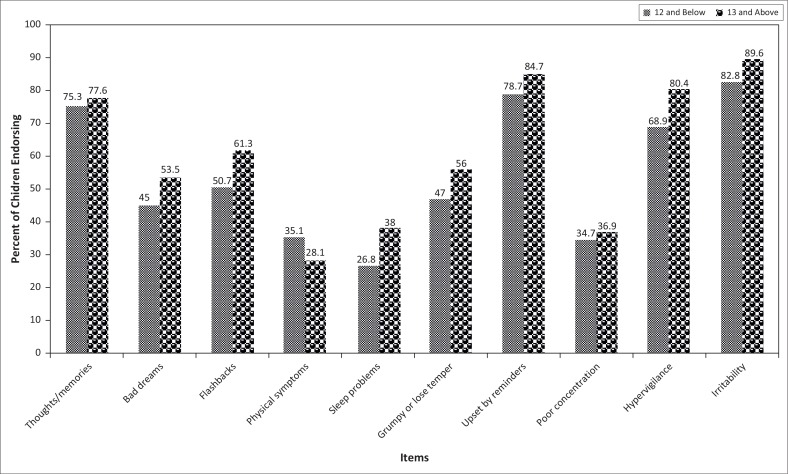
Percent of learners endorsing each item on the scale.

Of the 484 learners who were requested to participate in the survey, 433 completed the CTSQ. Of these, 429 had complete demographic data and responded to all the questions, and so, these were the questionnaires used for the analysis. Broken down by age, there were 134 (31%) learners aged 12 and below and 295 (69%) aged 13 and above. The researchers found it more informative to analyse the data by age category than by whether the learners were in primary or secondary school. Generally speaking, in the majority of cases, learners aged 12 and below would still be in primary school whilst those aged 13 and above would normally be in secondary school. The distribution of the learners by gender and age is shown in Table 1. All ages, starting at 9 years, were represented fairly well. One of the researchers’ aims was to see the extent to which younger learners were affected differently by the floods compared to older learners. The researchers therefore plotted each question on the CTSQ in order to compare by age category the proportion of the learners responding ‘yes’ to each of the items. Figure 1 thus indicates the percentages of learners endorsing each of the items, by age category. Across almost all the items on the CTSQ, the proportion of older learners responding with ‘yes’ were consistently higher than for younger learners with a ‘yes’ response. The only question concerning which younger learners had a higher proportion responding with ‘yes’ (35.1%) compared to the older learners (28%) was the question about having physical symptoms when reminded of the floods.

**TABLE 1:** Descriptive statistics of Child Trauma Screening Questionnaire scores by age and gender.

Gender	Age category	*N*	Mean	Standard deviation
Female	12 and below	90	5.37	2.14
	13 and above	181	6.33	2.21
Male	12 and below	44	5.43	2.14
	13 and above	114	5.84	1.94

According to Kenardy *et al.* (2006), a cut-off score of ≥ 5 would provide the optimum predictive value for PTSD screenings. Table 1 indicates the mean scores and standard deviations of the learners by age category and gender. The mean scores for all the groups of learners are greater than five, indicating that, as a whole, the learners exhibited symptoms of trauma.

**FIGURE 2:**
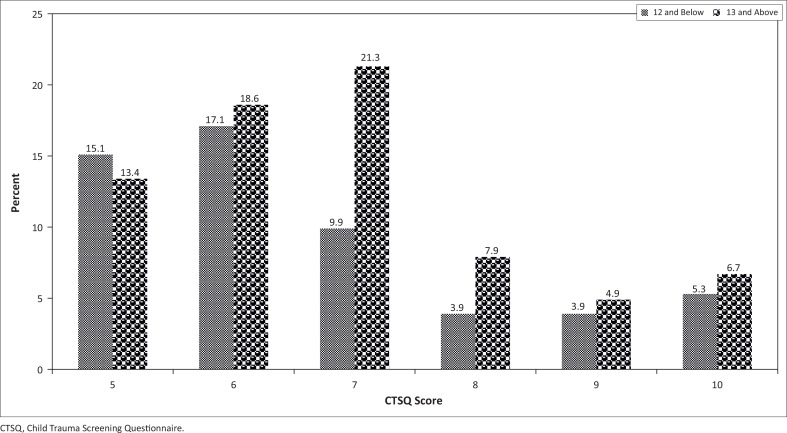
Distribution of learners with Child Trauma Screening Questionnaire scores of 5 and above, by age category.

Figure 2 indicates the percentage of learners with a CTSQ score of five and above. In total, 55.2% and 72.8% of the younger and older learners, respectively, reported experiencing symptoms of trauma from the floods 2 years after the event. These percentages are quite high and are a cause for concern to the researchers.

**FIGURE 3:**
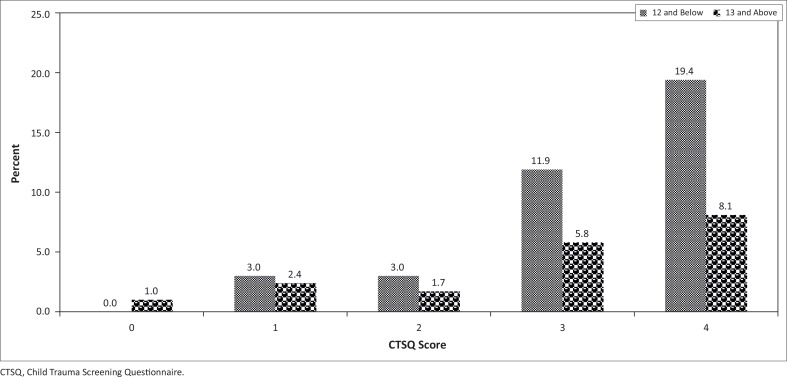
Distribution of learners with Child Trauma Screening Questionnaire scores below 5.

In contrast to the percentages shown in Figure 2, the distribution shown in Figure 3 is that of children with CTSQ scores below 5. These are the children with scores in a range that is not worrisome. The percentages were quite low, especially for children with CTSQ scores below 3.

## Testing for differences in the Child Trauma Screening Questionnaire means

A two-way analysis of variance was conducted to determine if there were differences in the CTSQ scores by age category and by gender as well as to test if there was an interaction between these two independent factors. The results indicated that there was a difference between the means of the two age categories: [*F*(1, 425) = 8.746, *p* = 0.003, partial η^2^ = 0.02]. There was no significance for either gender or the interaction between age and gender. The fact that the mean of the older learners was significantly higher than that for the younger learners might suggest that older learners were affected more by the floods than younger learners. It could also be that they remembered more of their experiences than did the younger children. The effect size for age was very small though (2% of the variance in CTSQ scores can be explained by age). Although the interaction between age and gender was not statistically significant, inspection of the means for the groups is ‘suggestive’ of a higher level of trauma amongst females aged 13 and above compared to their male counterparts.

## Discussion

The results of the CTSQ are quite disturbing as they suggest that, 2 years after the event, learners were still experiencing symptoms of trauma. Both age categories of learners had a mean score greater than five, suggesting that the effects of the floods were still lingering at worrisome levels. The older learners tended to have been affected more than the younger ones. They were also affected in higher proportions than the younger children. This is quite disturbing, given predictions of continued future occurrences of floods in this region (Lukamba 2010). With children being amongst the most vulnerable when it comes to natural disasters such as floods (Chitiyo *et al.* 2008), it behoves us to examine the nature of the impact in order to identify effective interventions designed to mitigate the impact. There is abundant research demonstrating that children’s daily living environmental experience affects their overall well-being (Berkman & Kawachi 2000; Marmot & Wilkinson 2006). According to the findings of this current study, 2 years after the floods, some children still experienced symptoms of post-traumatic stress disorder. This is consistent with previous reports suggesting that children affected by natural disasters usually experience psychological scarring, persistent emotional trauma, somatic complaints, re-experiencing, arousal, disruptive behaviour as well as depression and disillusionment (Chitiyo & Chitiyo 2009; National Child Traumatic Stress Network n.d.; Shibley 2010).

This study is not without its limitations though. Firstly, and most importantly, all the data regarding PTSD in this study are premised on the ability of the children to recollect events that happened 2 years prior to the administration of the CTSQ. The reliability and accuracy of the students’ responses may thus be questionable. Secondly, depending on the expectancy of the respondents about the potential benefits they might have received from the study, some students might have embellished their responses to make it seem as if they ‘suffered’ more than they really did.

## Implications for practice and recommendations

Though not surprising, what is particularly worrisome about the findings of the current study is that, 2 years after the floods, the majority of the children (i.e. 55.2% and 72.8% of younger and older learners, respectively) reported experiencing symptoms of trauma. Given the magnitude of this problem and the associated long-term sequelae, one would anticipate that psychological or emotional support would have the top priority amongst the services provided to the affected children. Unfortunately, this does not appear to have been the case as indicated by one of the principals who stated that services such as counselling were almost non-existent in both school and community. This might be because of a lack of professionals qualified to provide such services. In 2004, the Ministry of Basic Education, Sports and Culture, reporting on the development of education in the country, stated that the country had been unable to provide adequately skilled human resources necessary for the development of its entire education system (Ministry of Basic Education, Sports and Culture 2004). In light of the above, it is imperative for the government to train school and community counsellors and/or psychologists who would be able to provide the necessary psychological and/or emotional support in the event of future floods or similar disasters. Apart from counsellors and psychologists, it is also necessary to train special-education teachers who may provide pedagogical expertise necessary to meet the educational needs of children experiencing emotional or behavioural disorders. It is, therefore, important for the government to consider programs that promote the social well-being of children in its response should floods happen in the future. This requires proactive planning on the part of government to ensure that the right structures such as security and emergency sanitary facilities are in place.

This study makes a contribution to the existing literature on children and disasters. Generally, screening for trauma is conducted in the immediate aftermath of disasters. This study shows that, in the absence of adequate services to cater for the psychological needs of children immediately following disasters, the effects on children may linger for long periods and, even so, in magnitudes that are not negligible.
